# Multi-Stability and Pattern-Selection in Oscillatory Networks with Fast Inhibition and Electrical Synapses

**DOI:** 10.1371/journal.pone.0003830

**Published:** 2008-11-27

**Authors:** Tiaza Bem, Pierre Meyrand, Pascal Branchereau, John Hallam

**Affiliations:** 1 Institute of Biocybernetics and Biomedical Engineering, Polish Academy of Sciences, Warsaw, Poland; 2 Centre de Neuroscience Intégratives et Cognitives, Université Bordeaux 1 and CNRS, UMR 5228, Talence, France; 3 Maersk Mc-Kinney Moller Institute, University of Southern Denmark, Odense, Denmark; Indiana University, United States of America

## Abstract

A model or hybrid network consisting of oscillatory cells interconnected by inhibitory and electrical synapses may express different stable activity patterns without any change of network topology or parameters, and switching between the patterns can be induced by specific transient signals. However, little is known of properties of such signals. In the present study, we employ numerical simulations of neural networks of different size composed of relaxation oscillators, to investigate switching between in-phase (IP) and anti-phase (AP) activity patterns. We show that the time windows of susceptibility to switching between the patterns are similar in 2-, 4- and 6-cell fully-connected networks. Moreover, in a network (N = 4, 6) expressing a given AP pattern, a stimulus with a given profile consisting of depolarizing and hyperpolarizing signals sent to different subpopulations of cells can evoke switching to another AP pattern. Interestingly, the resulting pattern encodes the profile of the switching stimulus. These results can be extended to different network architectures. Indeed, relaxation oscillators are not only models of cellular pacemakers, bursting or spiking, but are also analogous to firing-rate models of neural activity. We show that rules of switching similar to those found for relaxation oscillators apply to oscillating circuits of excitatory cells interconnected by electrical synapses and cross-inhibition. Our results suggest that incoming information, arriving in a proper time window, may be stored in an oscillatory network in the form of a specific spatio-temporal activity pattern which is expressed until new pertinent information arrives.

## Introduction

Multi-stability of a dynamic system consists of the ability to express, for a given set of parameters, multiple stable states and to switch between these states in response to some external transient input. A few decades ago, the discovery of bi-stable cell properties (plateau activity) transformed understanding of the operation of the neural cell (see review [1]) as well as neural network operation [2]–[5]. More recently, studies in computational processes in non-oscillatory networks gave rise to the concept of a binary memory switch, where transient inputs can turn a plateau like activity on or off in a sub-set of cells within the network [6]–[12]. In the present study, we are interested in multi-stability of oscillatory networks generating rhythmic output. Bi-stability of in-phase (IP) and anti-phase (AP) solutions was first found in a half-center network model consisting of two inhibitory neurons with slow synaptic kinetics [13]. Such bi-stability does not necessarily require slow synaptic transmission and, indeed, it was also found when fast synaptic inhibition was combined with electrical coupling in similar network models [14]–[16]. Bi-stable behavior of a 2-cell inhibitory network has also been confirmed in dynamic clamp experiments on hybrid networks consisting of biological neurons of different intrinsic properties [15], [17].

Instantaneous reconfiguration of activity patterns by brief signals is potentially important for network operations, but the conditions and robustness of switching in a multi-stable oscillatory network still remain unknown. Here we analyze switching between patterns in a model network comprising relaxation oscillators interconnected by fast inhibitory synapses and electrical coupling. A relaxation oscillator is a model of a cellular pacemaker, commonly used to describe the slow envelope of membrane potential oscillation in bursting neurons (for example [18]). Also, in a short duty cycle regime (i.e. if a cell exerts synaptic action over a short part of the cycle), it is applicable to spiking neurons, in which an intrinsic regenerative mechanism is fast compared to recovery variable time scale [16]. Interestingly, moreover, relaxation oscillators are formally analogous to firing-rate models of excitatory neural network activity with slow negative feedback, like synaptic depression or cellular adaptation. Such population firing-rate models are used to study the bursting activity of populations of neurons which by themselves do not have pacemaker properties, as for example CPG networks in the developing spinal cord [19]. Moreover, if reciprocally interconnected via inhibitory subpopulations such network models serve for study of neural competition in such phenomena like binocular rivalry, perceptual bistability [20]–[22] or perceptual decision making [23].

In this paper we are interested in switching between in phase (IP) and anti-phase (AP) states in fully-connected homogenous networks of 2, 4 and 6 relaxation oscillators of short duty cycle. Our goal was to understand how switching rules, i.e. polarity, intensity and phase of stimulus producing a given switch, found for a 2-cell network can be generalized to a larger network. Although due to symmetry of the system one might expect that switching between IP and AP behavior will occur within the same window of the oscillatory cycle independently on the network size it was not clear whether the intensity of such switching stimuli remained the same. Indeed, increasing the size of a fully-connected network requires scaling of coupling parameters such that if total conductance of a single cell is kept constant synaptic coupling between any two cells decreases. This in turn may affect the basin of attraction of the IP or AP pattern and therefore efficacy of a switching stimulus of a given intensity.

Moreover, networks of larger size can generate several distinct AP patterns which is not the case in a 2-cell network. Here, our goal was to test whether properties of stimuli producing switching between AP patterns can be encoded in the resulting pattern of activity. This would not only provide a mechanism for storing signals incoming to the network in the form of a given activity pattern, as in models of working memory and line attractors, but also, since switching should occur only in specific time windows of the oscillatory cycle, offers an additional dimension of encoding.

Finally, a large inhibitory network in addition to IP and AP behaviors may express a multiplicity of other stable states, some of which may co-exist with IP and AP behaviors in some parameter domaines. If so, in addition to a transition between IP and AP switching from IP (or AP) to other states would be possible. Therefore, in order to explore and eliminate this possibility, we examined how the occurrence of multi-stable patterns depends on network size and determined an invariant parameter space for exclusive co-existence of the IP and AP states.

We first find a domain of coupling parameters in which only IP and AP patterns coexist independent of the network size. Thereafter we compare properties of stimuli producing switching between the patterns in networks of different sizes, for the same set of coupling parameters. We show that increasing the number of cells does not alter the properties of switching stimuli. Moreover, in the 4 or 6 cell network switching between different AP patterns was possible, the resultant AP pattern being completely determined by the profile of the switching stimulus, i.e. by the distribution of depolarizing and hyperpolarizing signals among cells. Finally we demonstrate that a firing-rate model network consisting of two oscillatory populations of excitatory cells interconnected by cross-inhibition and electrical coupling expresses switching between patterns according to rules similar to those found for two relaxation oscillators.

## Results

**Figure 1:**
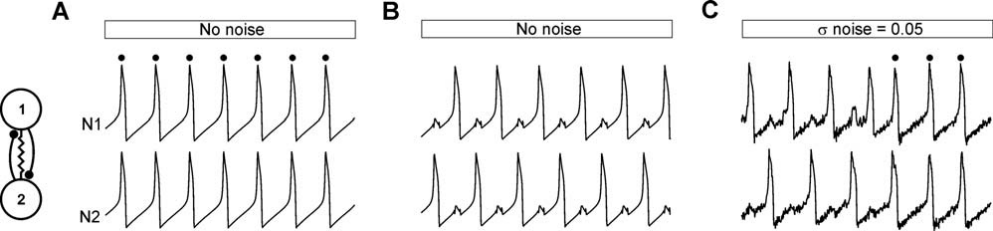
Bi-stability in model 2-cell networks. Networks consist of two cells interconnected by electrical synapses (resistor symbol in network diagram) and instantaneous reciprocal inhibitory synapses (solid lines with dots in network diagram). A. Model network consisting of relaxation oscillators expresses in-phase (IP) (A), or anti-phase (AP) (B) activity patterns for the same set of synaptic parameters. Introducing stochastic current input to both cells produces spontaneous transition between AP and IP (see black dots) patterns (C). Abbreviations: N relaxation oscillator model neuron. Parameters: *g^syn^* = 0.032, *g^el^* = 0.18, σ_noise = 0.05 (C).

**Figure 2:**
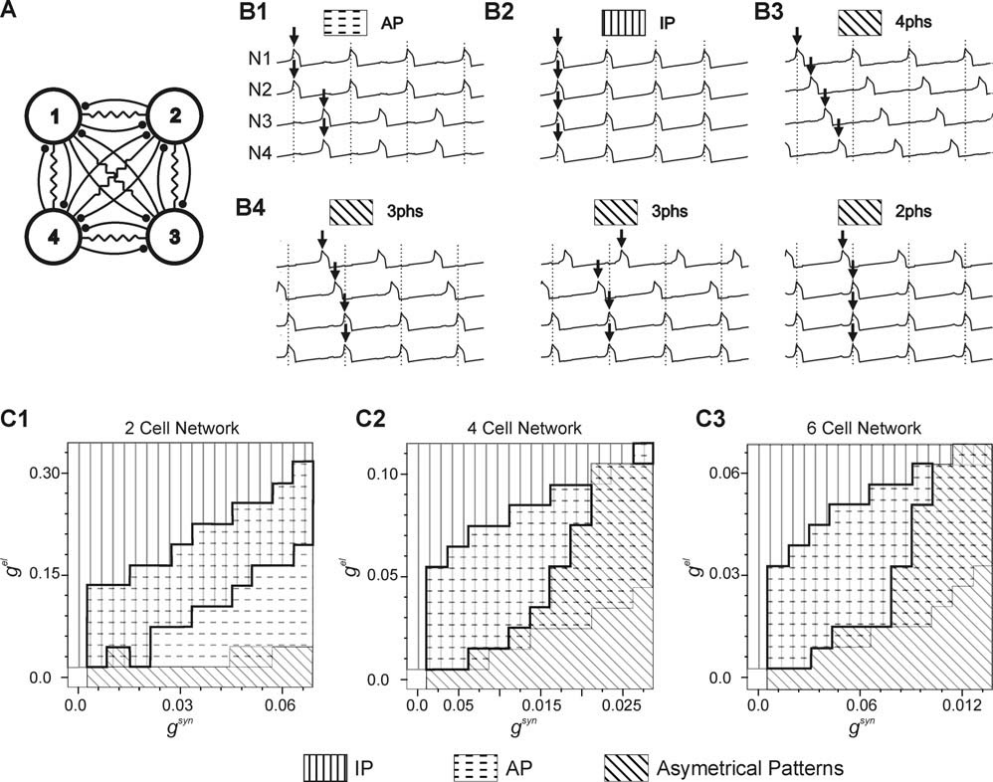
Multiple stable activity patterns in 2-, 4- and 6-cell network. A. Wiring diagram of 4-cell oscillatory network with full synaptic connections. B. Examples of various oscillatory behaviors expressed by the 4-cell network. Two symmetrical patterns: AP (B1) and IP (B2) are characterized by identical trajectory of all network members. Asymmetrical patterns characterized by different cell trajectories consist of 4 phase (4 phs, B3) or 3 and 2 phase behavior (3 phs, 2 phs respectively, B4). Notice the synergic group of 2 or 3 cells in B4. C. Occurrence of activity patterns as a function of synaptic strength in 2- (C1), 4- (C2) and 6-cell (C3) network. Parameters: *g^syn^* = 0.014 (B), *g^el^* = 0.06 (B1–B2), 0.01 (B3–B4), σ_noise = 0.005, independent identically distributed Gaussian for each 0.2 unit integration step in B and C.

Bi-stability of a 2-cell inhibitory network is a robust phenomenon. It has been demonstrated in modeling studies either for the slow inhibitory synapses alone [13] or for fast inhibition combined with electrical coupling [14]–[16] and, moreover, can easily be found in hybrid networks in which biological cells from snail ganglion [15] or cortical slices [17] are interconnected by a dynamic clamp system. In order to illustrate bistability of a 2-cell network we use model cells (relaxation oscillators) interconnected by fast inhibition and electrical synapses. In the model, IP (Fig. 1A, synchronous spikes are indicated by black dots above recordings) or AP (Fig. 1B) behavior is expressed for the same set of network parameters and switching between the two patterns can occur spontaneously if noise of sufficient amplitude is introduced to the network (Fig. 1C). As mentioned above, bistable behavior of a 2-cell model network has been already described [16]. We re-calculate here the occurrence of patterns in such a network (Fig. 2C1) in order to compare it with the behavior of larger networks (Fig. 2C2–3). In the 2-cell network, three types of activity pattern can be expressed depending on coupling strength: IP, AP and almost-in-phase (AIP). In contrast to IP and AP patterns which are symmetrical (cells' trajectories in the phase plane are identical), the AIP pattern is not symmetrical (trajectories of the cells differ) and consists in two cells' active phases expressed with the phase shift Φ<0.5 (see [16]). Note that the AIP pattern is expressed if oscillations underlying spiking are in a relaxation regime (the recovery variable time constant is large comparing to the membrane time constant); otherwise, instead of AIP, the AP pattern is present, as in a network consisting of reciprocally inhibitory integrate-and-fire cell models. The occurrence of the three patterns as a function of the inhibitory and electrical synaptic strength is shown in Figure 2C1. For inhibition alone, only the asymmetrical pattern is stable (see oblique line area, Fig. 2C1). Increasing electrical coupling produces a transition from this pattern to the symmetrical AP pattern (phase shift Φ = 0.5) (see horizontal dashed line area, Fig. 2C1). Further increase of the electrical coupling leads to the appearance of the IP pattern (see vertical line area, Fig. 2C1), which coexists with AP in some parameter domain (underlined surface Fig. 2C1).

The next larger network with a similar symmetry to the 2-cell network is a 4-cell network fully connected by inhibitory and electrical synapses (Fig. 2A). For this (and the larger) network we used intrinsic cell parameters identical to the 2-cell model network but scaled the synaptic conductance to maintain constant the total synaptic conductance of a single model cell. In other words, assuming network size equal N, a total electrical or inhibitory coupling between a given cell and the remaining N-1 cells was made independent of N.

With increasing network size, the number of stable patterns expressed increases. Indeed, in the 4-cell network, 4 stable asymmetrical and 2 symmetrical patterns were found in the presence of a relatively large amplitude noise input to the network (Fig. 2B). Like the 2-cell network, the 4-cell network expressed symmetrical AP (Fig. 2B1), and IP behavior (Fig. 2B2). Among asymmetrical patterns we found a 4 phase pattern in which all 4 cells fire at different phases within the cycle (Fig. 2B3), and 3 and 2 phase patterns in which synchronous firing occurs in groups of 2 or 3 cells (Fig. 2B4). Interestingly, the emergence of patterns seems to follow the same principle as in the 2-cell network (compare Fig 2C1 and C2). Indeed, with dominating inhibitory coupling, only asymmetrical patterns were expressed (see oblique line area, Fig. 2C2) whereas introducing electrical coupling led first to the appearance of AP and then IP behaviors (horizontal dashed and vertical line areas, respectively, Fig. 2C2) until, for a sufficiently strong electrical coupling, only the IP pattern remains. Importantly, the IP and AP patterns coexist in a similar subspace of coupling parameters as in the 2-cell network (compare underlined surfaces, Fig. 2C1 and C2). Finally, increasing the number of cells in the network to 6 did not change the qualitative distribution of pattern occurrence in the two dimensional parameter space (compare Fig. 2C1, C2 and 2C3).

**Figure 3:**
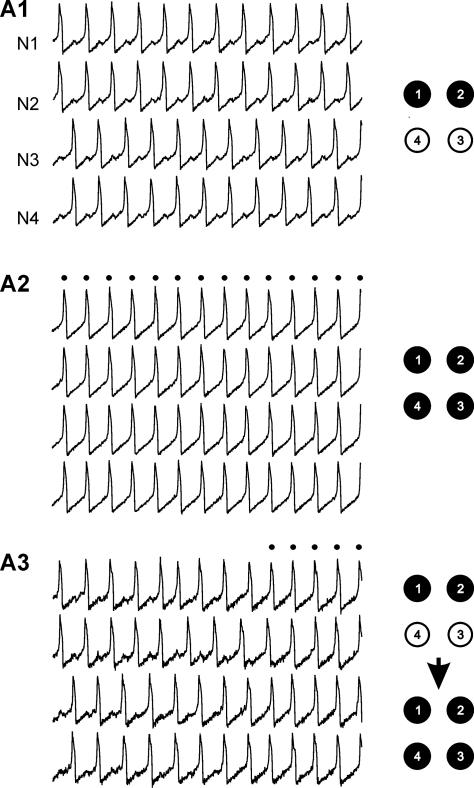
Bi-stability in 4-cell fully-connected model network. For a given parameter set the network either is divided into 2 groups of synergic cells (N1,2 in black and N3,4 in white) oscillating in AP (A1) or expresses synchronous activity (A2) where all the spikes occur simultaneously from N1 to N4 (see dots and scheme where all the cells are black). Spontaneous switching between these two modes of activity occurs due to the stochastic input signal (A3) (see transition from upper network scheme to bottom one). Notice increase of noise amplitude in A3 compared to A1 and A2. Parameters: *g^syn^* = 0.014, *g^el^* = 0.06, σ_noise = 0.025 (A1, A2), 0.05 (A3).

In the next section we will investigate transitions between these patterns. As illustrated in Figure 3, such transitions may occur spontaneously due to stochastic inputs. Indeed, with a low level of noise, the 4-cell network expresses either AP (Fig. 3A1) or IP (Fig. 3A2) patterns which persist an arbitrarily long time, while increasing the noise amplitude provokes spontaneous switching within a short time interval (Fig. 3A3).

**Figure 4:**
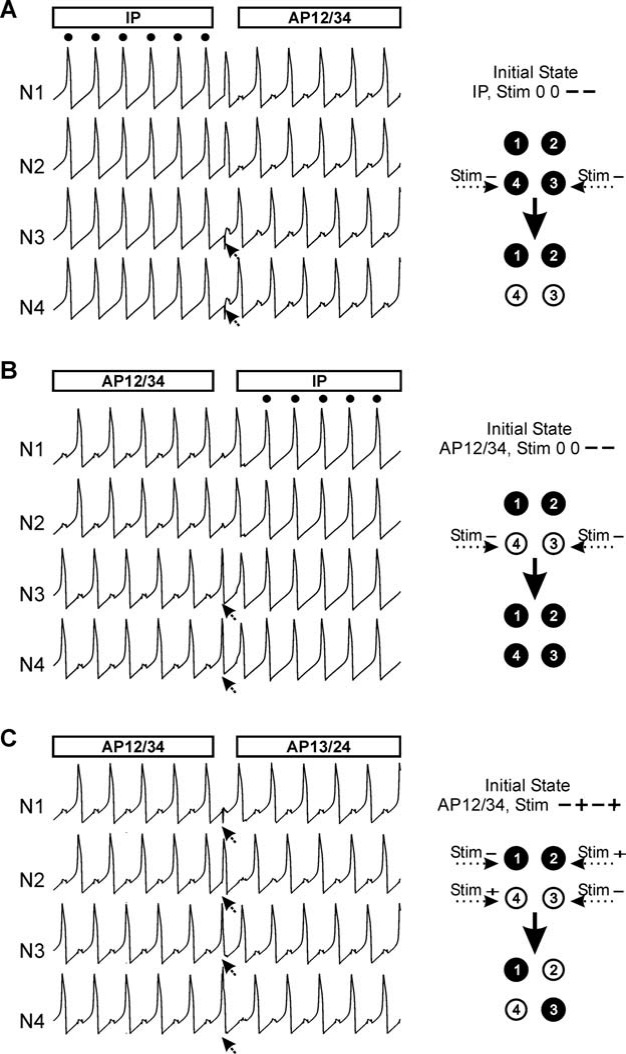
Transition between two stable states may be evoked by different types of stimuli. A. Delivery of transient hyperpolarizing stimuli to N3 and N4 (see arrows) during ongoing IP activity produces a sudden transition to AP behavior in which cells N3 and N4 oscillate out of phase with cells N1 and N2. This transition is schematically represented on the right panel. The profile of stimulus (here Stim “0 0 − −”) is encoded in a series of symbols corresponding to stimulus occurrence (0 no stimulus, + depolarizing stimulus, − hyperpolarizing stimulus) delivered to cell N1, N2, N3 and N4 respectively. B. The same type of stimulus (see right panel) during ongoing AP activity may produce the re-establishment of IP behavior. In this case the initial state is defined as AP12/34 indicating antagonistic activity of two groups of cells: N1, N2 and N3, N4, respectively. C: Stimulation of all cell members with the stimuli of mixed polarity (see right panel) may produce a transition between different AP patterns. Compare initial state AP12/34 with the resulting AP13/24 pattern. Parameters: *g^syn^* = 0.014, *g^el^* = 0.06, stimulus intensity = 0.4.

We will now consider non-spontaneous switching evoked by extrinsic stimuli. Since the network is oscillating, one may expect that the impact of a given stimulus will be phase-dependent. Therefore, we study not only the effect of polarity and intensity of a stimulus but also its efficacy depending on the time of delivery within the cycle. This is tested with respect to three types of switching: from IP to AP (Fig. 4A), from AP to IP (Fig. 4B) and between different AP patterns (Fig. 4C). In all these cases, initial and resultant states of the network are illustrated in diagrams showing clusters of synchronous cells (group of cells either black or white). For example, a switch from IP to AP is illustrated as a transition between four black cells to two black and two white cells (right panel, Fig. 4A). Here the stimulus profile is described as “0 0 − −” indicating that cell N1 and N2 do not receive any input whereas a transient hyperpolarizing input is delivered to cells N3 and N4. An identical stimulus profile evokes a reverse switch between AP to IP (Fig. 4B).

Notice that the AP pattern shown in figure 4A–B is not the only possible AP behavior of the network. Indeed, an AP state consists in a division of the network into 2 groups of synchronous cells, the groups oscillating in anti-phase. Therefore in the 4-cell network there are 3 possible such divisions: AP12/34, AP13/24 and AP14/23. Here switching from AP12/34 to AP13/24 was evoked by delivering hyperpolarizing inputs to 2 cells (N1 and N3) and depolarizing inputs to the 2 remaining cells (N2 and N4) (see stimulus profile “− + − +”, Fig. 4C).

**Figure 5:**
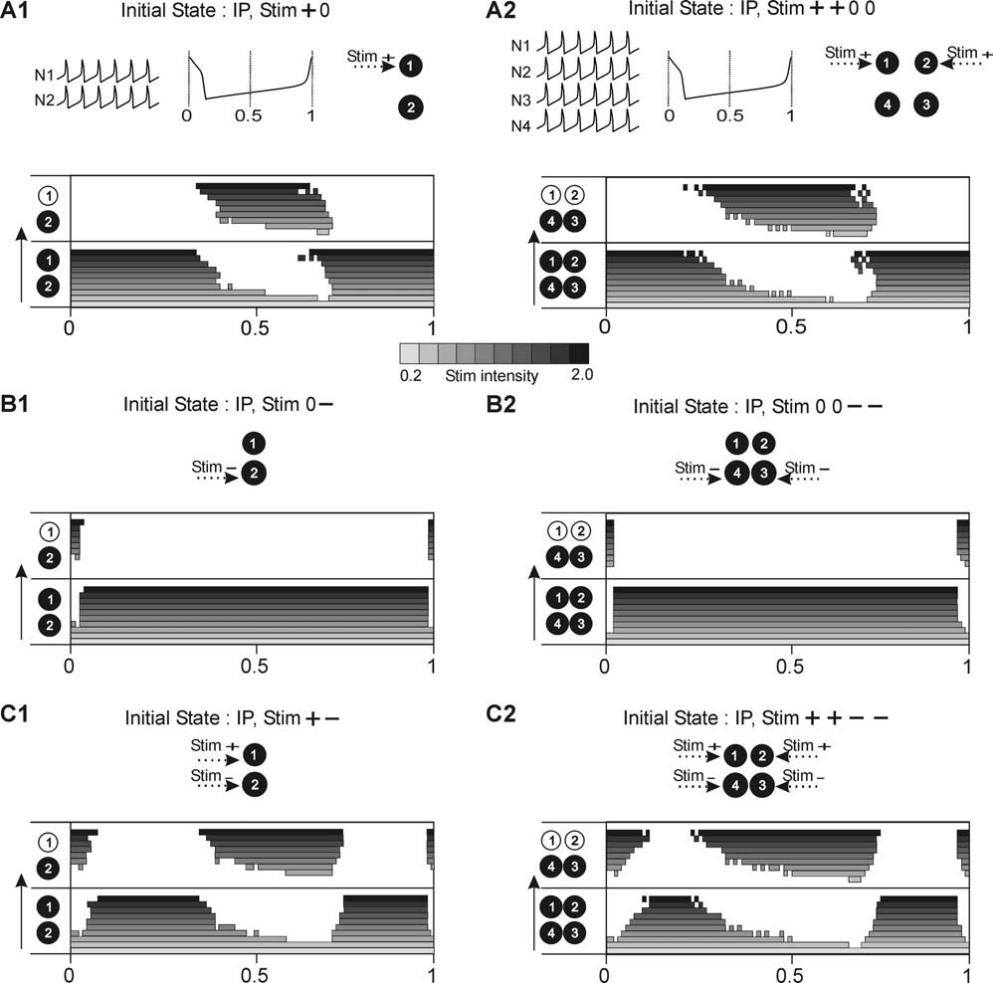
Time windows of susceptibility to switching from IP to AP pattern in 2- and 4-cell network. A. Shown is ongoing in-phase activity, sketch of the oscillatory cycle and diagram of stimulus profile in the 2-cell (upper panel, A1) and 4-cell (upper panel, A2) networks. In each bottom panel switching diagrams are shown for different intensities of depolarizing stimulus, represented by different intensities of gray (see scale). The diagram is divided into the bottom part, representing the initial state, and the top part representing the resulting pattern. For each current intensity, stimuli of duration 0.3 time units are delivered in different phases of the cycle with a step of 0.2 time units (cycle period is c. 20 time units for all patterns shown). The stimulus of lowest intensity does not produce a switch in any phase of the cycle. This is indicated by a continuous horizontal bar in the bottom part of the diagram (A1, A2). Increasing the stimulus intensity results, in some phases of the cycle, in an effective stimulation which is indicated by a gray bar in the upper part of diagram. Notice a similar mid-cycle window in the 2-cell (A1) and 4-cell (A2) networks. B. With an hyperpolarizing stimulus switching is effective only during the firing phase of the stimulated cell for the 2-cell (B1) and 4-cell (B2) networks. C. Stimulus of mixed polarity (see upper panel) evokes switching both in mid-cycle and in vicinity of cells' firing phases in 2- (C1) and 4- (C2) cell networks. 2- and 4-cell network parameters are the same as in figures 1A and 2B1–B2, respectively.

All examples illustrated in Figure 4 show successful switching. As we shall see in the next sections, they occurred because stimuli were applied at the proper time within the cycle. Indeed, as shown in [15], in the 2-cell network switching from IP to AP was possible, for example, when a depolarizing stimulus was applied to one of the cells at a time halfway between two successive spikes. Here we explore this switching again by delivering depolarizing stimuli of different intensity (see scale bar, top Fig 5) to one of the cells (see Stim “+ 0”, Fig. 5A1) while the network is expressing IP behavior (initial state). In order to explore the phase dependence of stimulus impact, we delivered it at different moments of the cycle from phase Φ = 0 to 1, corresponding to the maxima of depolarization of the consecutive spikes of cell N1 (see central inset Fig. 5A1). For low intensity stimulus, the initial state of the network remains unchanged independently of the timing of stimulus delivery within the cycle (see lighter grey bar, Fig. 5A1). Increasing the intensity of stimulus provided a time window around the middle of the cycle where switching to the AP behavior occurred (Fig. 5A1). Further increase stimulus intensity did not substantially alter this window. Interestingly, the application of analogous stimuli (i. e. depolarizing stimulus to 50% of cells) for a 4-cell network (see Stim “+ + 0 0”, Fig. 5A2), revealed a very similar window for switching (Fig. 5 A2).

By contrast to depolarizing stimuli, which offered relatively large switching windows (up to half of the cycle), hyperpolarizing stimuli distributed among 50% of cells produced switching only if delivered in a very narrow time window during firing of stimulated cells in both the 2-cell (Fig. 5 B1) and the 4-cell network (Fig. 5 B2). Finally, simultaneous application of stimuli of opposite polarities, each now delivered to 50% of cells (see stimulus profiles, Fig. 5C1 and C2), resulted in switching windows which combined the main features of the two types of switching window corresponding to the singe stimulus polarities (Fig. 5 C1, C2). Indeed, with this type of stimulus, switching from IP to AP behaviors was produced in the middle part of the cycle as well as around the spike generation phase (i.e. Φ = 0 and 1). (Note that although the compound window is a qualitative sum of components it is not a simple linear combination.)

**Figure 6:**
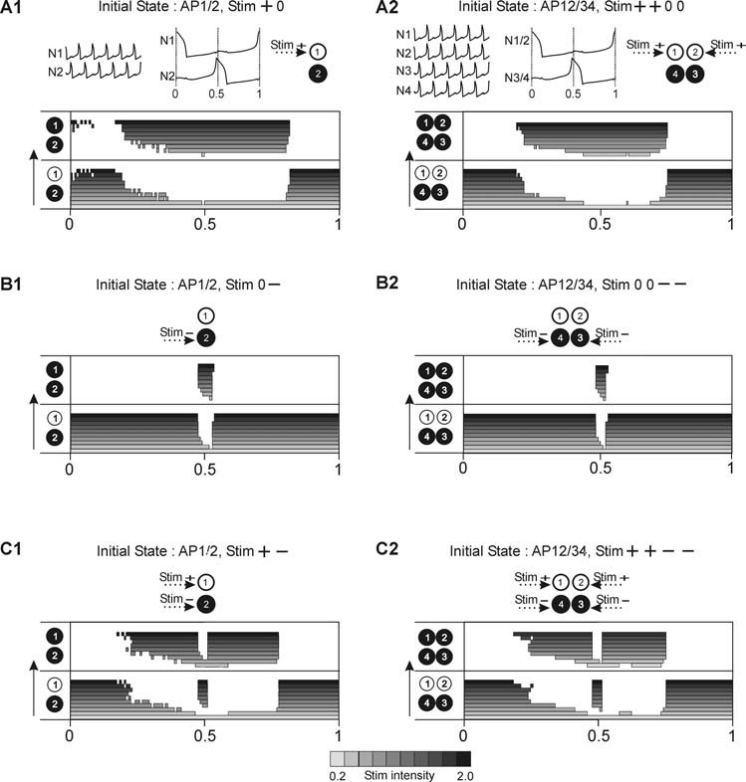
Time windows of susceptibility to switching from AP to IP pattern in 2- and 4-cell network using homogenous stimulus to synergic 50% of cells. A1–2. The schematic representation in the upper panel is the same as described in Fig. 5A. Depolarizing stimulus delivered to cell N1 (A1) or cells N1, N2 (A2) evokes a switch if applied in a large mid-cycle window. B1–2. Hyperpolarizing stimulus delivered to cell N2 (B1) or cells N3, N4 (B2) evokes switching only in a narrow window around Φ = 0.5. C1–2. Mixed polarity stimulus delivered to all network members has a similar effect as depolarizing stimulus except around Φ = 0.5 where switching fails. Network and stimulus parameters as in Fig. 5.

We will now consider reverse transitions, i.e., switching from the AP to the IP pattern. When the network is in AP mode it is divided into two groups of cells (see top panels in Fig. 6A1–A2); this asymmetry increases the number of distinct stimulus types.

First, if we apply a stimulus of a given polarity to just one of the two groups (see stimulus profiles in Fig. 6A, B), switching windows show features similar to those which characterize transitions from IP to AP behavior. Indeed, in both 2-cell and the 4-cell model networks, positive stimuli delivered to one group of synergic cells evoked switching to the IP behaviors when applied in the middle of the cycle (Fig. 6A1, A2). Here the window for switching is wide, occupying approximately half of the cycle, similar to the analogous window for IP to AP transitions (see Fig. 5A1, A2). Furthermore, negative stimuli delivered to just one group of cells are likewise effective only when applied during spiking of the stimulated cells (Fig. 6B1, B2) as was the case for IP to AP switching (Fig. 5B1, B2). Notice however, that the window is now located at Φ = 0.5 since the stimulated group is now phase shifted by 0.5 with respect to the reference cell in the initial AP behavior. Finally, when stimuli of opposite polarities were delivered to the two antagonistic groups of cells, switching to IP was still evoked in a large window located around the middle of the cycle (Fig 6 C1, C2). However, at Φ = 0.5 these transitions were not possible: here, delivery of the stimuli produced simultaneous exchange of the phases of the two groups - spiking cells became silent and silent cells became spiking. This resulted in a reset of the ongoing activity (not shown) but did not change the AP pattern.

**Figure 7:**
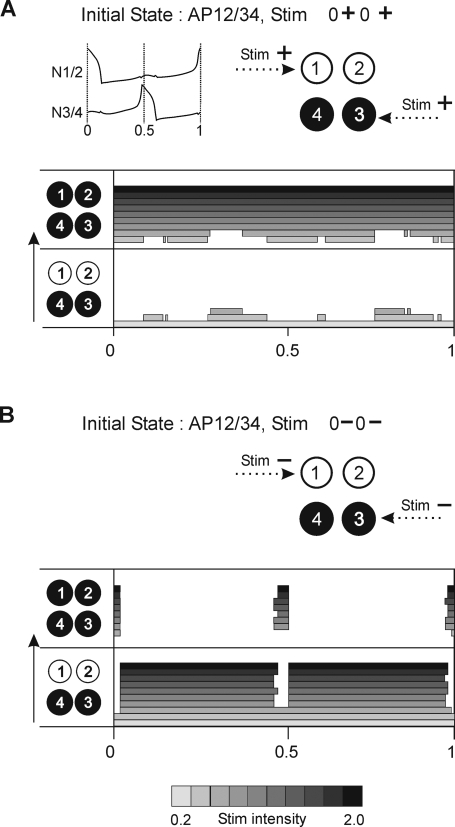
Time windows for AP to IP switching with homogenous stimuli delivered to 50% of cells in each synergic group. A. Depolarizing stimulus produces switching independently of the time of its delivery. B. Hyperpolarizing stimulus is effective only if applied during firing phases. Network and stimulus parameters as in Fig. 5.

Second, stimuli of a given polarity can be delivered to cells belonging to both antagonistic groups such that only 50% of network members receive input (see stimulus profiles, Fig. 7). If depolarizing, such a stimulus is very effective and produces switching to the IP behavior independently of the phase of delivery (Fig. 7A). If hyperpolarizing, its impact is again restricted to phases when one of stimulated cells fires (Φ = 0, 0.5, 1, Fig. 7B). (For a mixed stimulus of this type, see below.) It must be noted that similar features are expressed by the 2-cell network if both cells are stimulated with the same polarity (data not shown).

**Figure 8:**
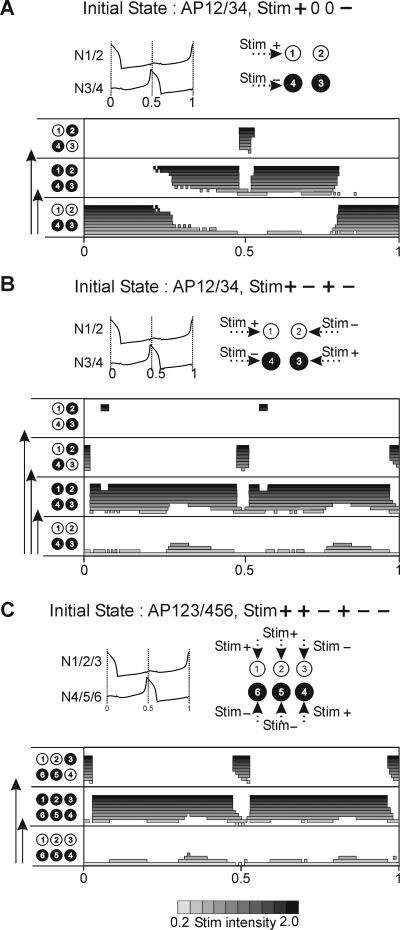
Mixed stimulus may produce switching between different AP behaviors in 4- and 6-cell network. A. Stimulus of opposite polarities delivered to antagonistic cells (50% of network members) may produce either switching to IP if delivered within a large mid-cycle window except Φ = 0.5 or switching to another AP pattern if delivered at Φ = 0.5. B. Mixed stimuli applied to the whole population with opposite polarities distributed among synergic cells (see upper panel) evokes switching to IP everywhere in the cycle except if delivered close to firing phases where switches to another AP pattern occur. The new AP pattern expresses the map of activity corresponding to the stimulus profile (AP13/24). Note that stimuli of very high intensity may produce AP with activity map different from both the stimulus profile and the initial AP map (see AP14/23). C. Qualitatively similar switching is found for the 6-cell network.

Third, stimuli of opposite polarities delivered to antagonistic groups, again such that only 50% of network members receive input, (see stimulus profile, Fig. 8A) evokes switching to IP behavior when applied in a large window around middle of the cycle, except at Φ = 0.5 (Fig. 8 A). At Φ = 0.5 a switch into a new AP pattern occurs (from AP 12/34 to AP 13/24) because both stimulated cells, belonging to different synergic groups, exchange their memberships: at Φ = 0.5 the spiking cell (N4) become silent due to hyperpolarizing stimulus whereas the silent cell (N1) generates a spike in response to depolarizing stimulus. Applying the same type of stimulus also to the remaining 50% of cells, in such a way that in each synergic group the cells received stimuli of different polarities (see stimulus profile, Fig. 8B), always produced switching to the IP pattern, except if applied during cells' spiking phases (Φ = 0, 0.5, 1) (Fig. 8B) where switching to another AP pattern took place (from AP 12/34 to AP 13/24). Indeed, in each spiking phase, a pair of cells receiving stimuli of opposite polarity and belonging to different synergic groups can exchange their memberships, as described above. Notice, importantly, that the distribution of delivered stimulus is now encoded in the resulting AP pattern. Indeed, cells which receive a stimulus of the same polarity will form new synergic groups (see N1, N3 and N2, N4, Fig. 8B). This results in a new division of the network which is equivalent to a new AP behavior (AP 13/24). Finally, it must be noted that for very high stimulus intensity, another AP pattern variant may emerge instead of the expected pattern (see top panel in Fig. 8B).

Although the 4-cell network reveals some general rules of encoding the stimulus profile as resulting pattern, the network is nevertheless insufficiently general. In particular, it is never possible to switch a majority (or minority) of a synergic group with a corresponding set of cells in the other group – because groups comprise just 2 cells (and since only 1 cell can be switched it is always 50% of a group). Therefore, to generalize the above encoding rules, we used a similar approach on the 6-cell network. Here, within each group of synergic cells consisting of 3 cells, 2 cells receive stimulus of the same polarity (see stimulus profile, Fig. 8C) and therefore are supposed to remain synergic in the resulting AP pattern. Interestingly, switching indeed occurs from AP123/456 to AP124/356 so that pairs (N1, N2) and (N5, N6) which received homogenous stimuli remained synergic. By contrast cells N3 and N4 switch their memberships because the signals they receive are of opposite polarity to the signals sent to the other group members. Moreover, time windows for switching to the new AP pattern were the same as in the 4-cell network: such switching occurred only if the stimulus was delivered during the cells' spiking phases; otherwise stimulus delivery resulted in full network synchrony (Fig. 8C). It should be stressed that in the 6-cell network the distribution of the applied stimuli again determines the resulting AP pattern (N1, N2 and N4 received depolarizing stimuli and N3, N5 and N6 hyperpolarizing inputs, therefore clusters 1, 2, 4 and 3, 5, 6 are formed).

**Figure 9:**
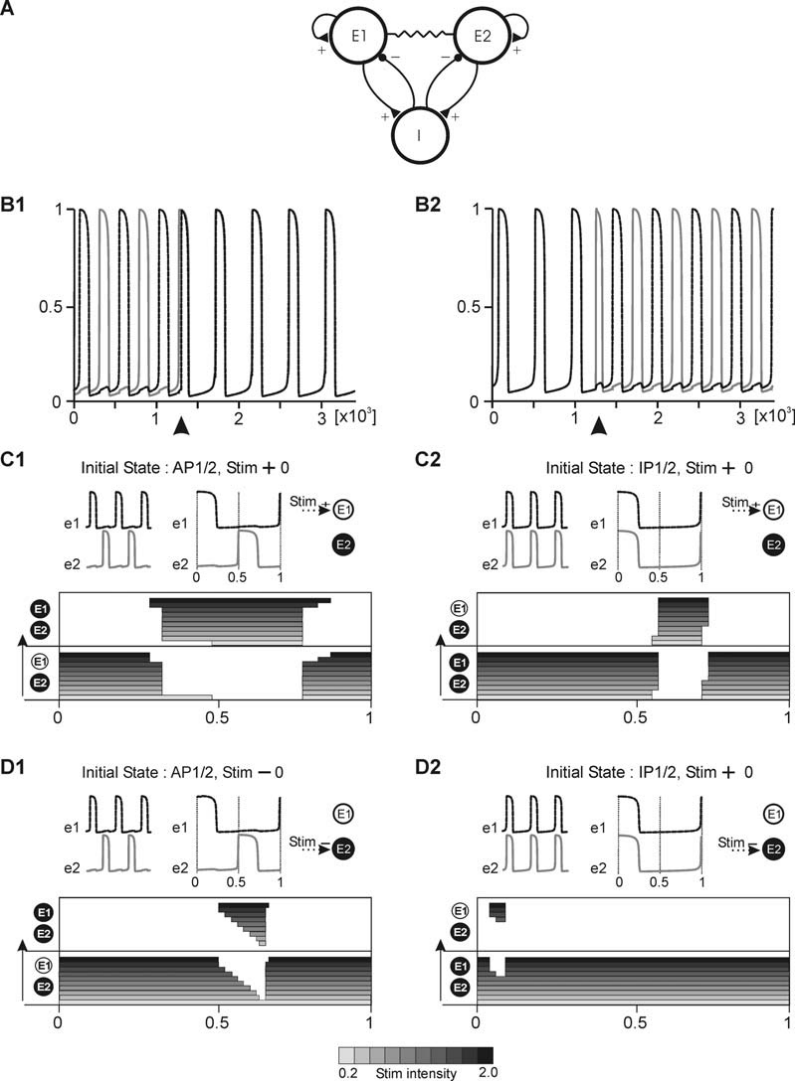
Bistable behavior of two excitatory populations interconnected by cross-inhibition and electrical coupling. A. Network architecture. Excitatory and inhibitory connections are represented by triangles and filled circles, respectively whereas resistor symbol represents electrical coupling. E1, E2 represents excitatory and I inhibitory populations. B. Time course of activity e1 (black line) and e2 (gray line) in populations E1 and E2. Transient input (arrow head) produces switching from AP to IP (B1) or vive versa (B2). Positive stimuli (C1, C2) are more efficient in switching than negative, which are restricted only to active phases (D1, D2). Parameters: *τ_e_* = 1, *τ_s_* = 250, *w* = 0.7, *V* = 0.17, *β* = 0.06, *i_tr* = 0.3, *g* = 0.075, *g_s_* = 0.025, *θ* = 0.3 *ks* = 0.05 *ke* = 0.05. *V_app_* = 0.1 (in B), 0.02–0.2 (in C and D, see bottom bar), stimulus duration = 10 time units.

As mentioned in the Introduction, there is a formal analogy between a relaxation oscillator and a firing-rate model of excitatory neural network activity with slow negative feedback [19]. We therefore tested whether our results are applicable to a model network consisting of two excitatory populations with recurrent connectivity, in which synaptic depression played the role of a slow process underlying oscillations. Both populations were connected by electrical synapses and projected to a common inhibitory pool which provided cross inhibition (Fig. 9A, see also Method). (It must be noted similar network behavior to that described below is expressed if populations E1 and E2 project to separate inhibitory pools.) As illustrated, such a model network expresses bistability of IP and AP patterns (Fig. 9B) and switching between these can be produced by transient inputs in similar time windows to those for a network consisting of relaxation oscillators (compare figures 9C1 and 6A1, 9D1 and 6B1, 9C2 and 5A1, 9D2 and 5B1). This also suggests that switching in larger networks consisting of many oscillatory units will be similar independently of whether a “unit” consists of a single cell or of a population of cells.

## Discussion

In the present study, we demonstrate that an oscillatory model network, consisting of more than 2 cells, fully interconnected with fast inhibitory and electrical synapses, generates a large variety of stable activity patterns, the number of which depends on the number of cells as well as on the coupling parameter domain (see Fig. 2). Moreover, for a given set of synaptic strengths, multiple stable patterns can be expressed and switching between them can be induced by a suitable transient external stimulus. In this paper, we focused on the parameter domain where only IP and different AP patterns co-exist. We generalized rules governing switching between IP and AP patterns by comparing behaviors of networks of different sizes.

It must be noted that whereas increasing the network size diminishes the strength of intra-network cell-to-cell connectivity – a condition necessary for exclusive coexistence of AP and IP patterns (see Fig. 2) – the intensity of external stimulus per cell, required for a given switch, remains the same (see Fig. 5 and 6). The other scaling, providing a constant cell-to-cell synaptic strength, may seem to be more intuitive. However, in this case inhibition and electrical coupling between a cell and the rest of the network become so strong with increasing network size that they produce a damping of the amplitude of oscillations and their eventual suppression (as previously described [16]). Note also that this latter scaling regime implies that cells can sustain an arbitrarily large total conductance, implausible given their morpho-functional limits.

**Figure 10:**
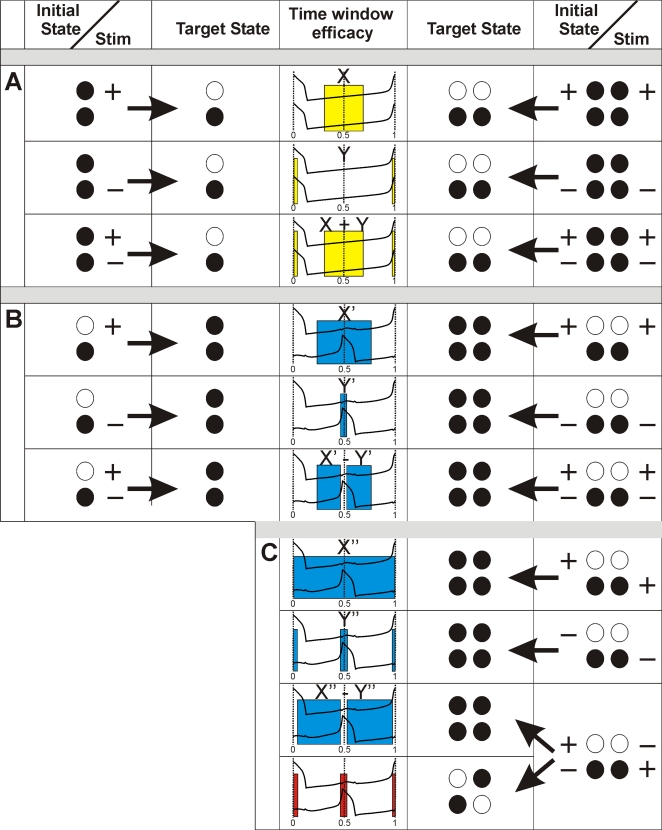
Rules of switching between multistable patterns depending on stimulus profile. (see Discussion).

The switching rules are illustrated in Fig. 10 where they are summarized for 2- and 4-cell networks. Assuming stimulus delivery to 50% of the cell population, transitions from IP to AP are produced by a depolarizing stimulus if it is applied within a relatively wide time window located at mid-cycle (type X window) (upper panel Fig. 10A). On the other hand, a hyperpolarizing stimulus can produce switching only by suppressing the spike of a cell and therefore this switching is very phase-specific (phases 0, 1, type Y window) (middle panel Fig. 10A). If these two types of stimuli are applied simultaneously to different sub-populations of cells, the resulting time window is the sum of the two windows corresponding to depolarizing and hyperpolarizing stimuli (type X+Y window) (bottom panel Fig. 10A).

Reverse transitions are more complex. Indeed, the network expressing AP behavior is divided into two antagonistic groups of cells and therefore distribution of stimuli between these two groups becomes important. Consider two possibilities.

First, assuming that only cells belonging to one of these groups are stimulated, a transition from AP to IP occurs if a depolarizing signal arrives within a large mid-cycle window (type X′) (upper panel Fig. 10B), or if a hyperpolarizing signal is applied in a very narrow window restricted to the cell firing phase (type Y′) (middle panel Fig. 10B). When stimuli of both polarity are distributed among the network cells, the resulting window is now not a sum (see bottom panel, fig 10A) but a difference between the two window types (Type X′–Y′) (bottom panel, Fig. 10B).

Second, if stimuli of the same polarity are delivered to members of different groups, transitions to IP also occur. Here, depolarizing stimuli produce switching independently of the time of delivery (Type X″) (upper panel, Fig. 10C), the impact of hyperpolarizing stimuli is again restricted to the firing phases of neurons (Type Y″) (middle panel, Fig. 10C) and mixed stimuli provide switching if delivered anywhere in the cycle except during firing phases (Type X″–Y″) (upper line, bottom panel Fig. 10C). Importantly however, during these firing phases the network switches from a given AP to a new AP pattern. The resulting AP pattern consists in a new division of the network into two new sub-populations of cells, corresponding to the distribution of hyperpolarizing and depolarizing stimuli among the network members (bottom line, bottom panel Fig. 10C). Therefore the information carried by the transient stimulus is now encoded in a given persistent spatio-temporal oscillatory pattern expressed by the network.

These results indicate that, within networks consisting of oscillatory cells interconnected by fast inhibitory and electrical synapses, reconfiguration of activity patterns may be evoked by transient input and the new configuration encodes a profile of the stimulus.

The idea of storing the memory of a transient stimulus in the form of a given spatial distribution of active cells has been used in the bi-stable model network expressing switching between global OFF and multiple ON activity states which was proposed as a model of working memory [12]. There is no OFF state in an oscillatory network since activity is always expressed as synchronous, or one of the different patterns of asynchronous, firing of neurons. Instead of switching between OFF and one of the multiple ON states (depending on a given subpopulation of active cells) such a network expresses transitions between IP and one of the multiple AP states (depending on a given division of the network into two parts), that is a transition between two rhythmic outputs of different frequencies. Therefore in both network types the average frequency of neurons can be altered by a transient stimulus. Apart from this similariry the switching properties of oscillatory networks are quite different from non-oscillatory networks expressing bi-stability. Indeed, the impact of a transient input on the ongoing network activity is phase dependent. Therefore the same incoming information will have different effect on network behavior (or have no effect at all) if arriving in different phases of the oscillatory cycle. Moreover, stimuli of the same profile (i.e. same polarity and distribution among the cells) can produce switching back and forth between IP and AP patterns. By contrast, a specific stimulus is required to switch ON or OFF the activity in the non-oscillatory network.

Furthermore, in the oscillatory network direct switching between different AP states is possible, whereas in the non-oscillatory network in order to establish a new ON state the network activity must be first reset to the OFF state. Finally, switching between states can be very fast within oscillatory network.

Beside the possible function of storing information as proposed in models of working memory, the multi-stability of oscillatory networks may also play an important role in functional reconfiguration of dynamical systems. For example in motor control, during ongoing activity of the CPG network it has been shown that the sensory information from the periphery as well as feedback originating from special senses (vision, audition, vestibular) may shape the motor output in a phase dependant way (see review [24]). These inputs continuously adjust the output of the network during a given motor task. However, when a sudden change is needed, for example a transition from one gait to another, it can be executed by a transient signal which changes instantaneously and simultaneously the phase relationships between all the network elements involved in the task. Interestingly, in the area of bimanual finger tapping two coordination patterns of different stability have been found ([25], [26]). In the search for principles of pattern generation in complex biological systems, a theory of self-organization in non-equilibrium systems including order parameters dynamics, stability, fluctuations and times scales has been proposed [27]–[29]. From such a perspective, multi-stability of neural networks appears as a commonly occurring natural phenomenon. Indeed, in accordance with the theory, it has been demonstrated that a transition between bi-stable patterns of finger tapping can be elicited in the human brain by transient transcranial magnetic stimulation [30]. Unfortunately, in that study no attempt was made to determine time windows for susceptibility of switching between patterns. Relaxation oscillators used in this study are basically models of the slowly oscillating envelope of bursting neurons, but in a short duty cycle regime may be also applicable to spiking neurons [16]. Factors that may contribute to differences between our idealized cell model and a real spiking neuron include non-relaxation intrinsic cell dynamics and slow synapses. How they influence switching in bi-stable network should be tested in further modeling studies. Interestingly, our preliminary results indicate that 2- and 4-cell networks consisting of IF cell models express qualitative features of switching windows very similar to those described for a network composed of relaxation oscillators.

Our results are also applicable to networks consisting of oscillatory circuits, which express periodic changes of cells' mean firing rate (Fig. 9). It seems likely that switching rules found for a given topology of connections between relaxation oscillators, like those for full connectivity presented in this study, could be also applicable for such a “network of networks”, independent of details of individual cells' or synapses' properties. This could further support a hypothesis that switching between multistable patterns can occur during gait control provided be GPGs networks which are known to consist of oscillatory units with a well-defined symmetry of connectivity [31], [32]. Our model requires, in addition to cross-inhibition, electrical coupling between oscillatory units to express AP pattern in the case of short duty cycle, as described previously [16]. Although such coupling has so far been described only in invertebrates' CPG, recent work suggests that it may also play an important role in mammalian CPG networks [33].

In summary, our data demonstrate that a given network operation can be altered without change of cellular or synaptic properties and with almost no activation time. A reconfiguration through neuromodulation has been shown to play an important role in shaping properties of a network's hardware elements in both developing and mature networks. However, as demonstrated by our data, in the multi-stable network, different stable rhythmic patterns can be selected by specific transient stimuli without any change of network topology, membrane or synaptic parameters.

## Materials and Methods

### The Cell Model

Cells in the model network are modeled as a set of first order differential equations, each cell contributing two state variables to the set: the instantaneous membrane potential (*V_i_*) and a slow recovery current (*W_i_*) dependent on membrane potential. The variables have the (non-dimensionalised) dynamics defined by equation 1–2.
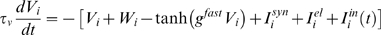
(1)

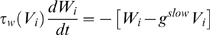
(2)


In equations 1–2 *g^fast^* determines the degree to which the instantaneous voltage-dependent current is N-shaped whereas *g^slow^* models the voltage-dependent activation function of the slow current. Synaptic transmission is instantaneous, with a synaptic current given by
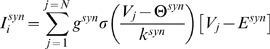
(3)where *g^syn^* is the maximal synaptic conductance, *V^i^* is the membrane potential of the the presynaptic cell *j*, *E^syn^* is the synaptic reversal potential, *Θ^syn^* represents the midpoint for synaptic activation and *k^syn^* the steepness of the synaptic activation function. The function *σ(x)* is defined as *σ(x) = 1/(1+e^x^)*.

Gap junction coupling is represented by
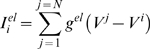
(4)where *g^el^* is electrical conductance. 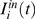 is externally injected input current. *τ_v_* and *τ_w_ (V_i_)* are the membrane time constant and the time constant of slow current dynamics, the latter depending on the membrane potential *V_i_*:
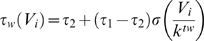
(5)with *τ_1_* and *τ_2_* specifying the minimum and maximum time constants and thereby determining the durations of the active and silent phases of the oscillator – and *k^tw^* quantifying the rate of voltage dependence.

The complete model defined by equations 1–4 has, therefore, three time constants, two membrane and two junction conductances, synaptic threshold and reversal potentials and two rate constants (the k parameters). In principle, the junction parameters can vary per junction while the neuron parameters may vary per neuron; for tractability in the current work these parameters are identical for all junctions and neurons respectively, with the following values: *E^syn^* = −4, *Θ^syn^* = 0, *k^syn^* = 0.02, *g^fast^* = 2, *g^slow^* = 2, *τ_1_* = 5, *τ_2_* = 50, *k^tw^* = 0.2, *τ_v_* = 0. 16.

The parameters have been chosen to model neurons with relatively steep synaptic onset and short duty cycle (i.e. short fraction of the cycle when the cell is depolarised above threshold and may exert synaptic action). With this choice of parameters, equations 1–5 may be considered a model of spiking neurons. In the study presented here, the only parameters varied from the defaults are the conductances *g^el^* and *g^syn^* of the electrical and inhibitory synapses.

The model has been implemented as a set of Matlab functions which compute the quantities defined by the five equations above and integrate the set of ordinary differential equations using Matlab's standard ode45 solver with the default tolerance parameter settings. Note that the external timestep used (0.2 units) is long compared to the internal timesteps available to, and typically employed by, the ode45 integrator. The external input currents 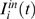 are assumed to be piecewise constant, also over intervals long compared to the internal integration timestep. The implementation has been compared to an independent realisation using the xpp tool and found to give identical results.

### Analysis Methods

The investigation of the oscillatory behaviours generated by networks of the type under study is time-consuming and has been automated. For a given choice of conductance parameters, the network dynamics are integrated from an initial state (see below). The first 30% of the simulation following the completion of any non-zero input signal is discarded to mitigate the effects of transients and the membrane potentials are computed at time points with an interval of 0.2 units. Given these values, an attempt (which may fails: typical causes of failure are chaotic network behaviour or very long period oscillation) is made to estimate a period of regular oscillation for the network by computing the positions of peaks in the autocorrelation of the signals and finding the highest common factor of inter-peak periods. If this calculation fails, the network is simulated further and the calculation repeated. If no period can be found with simulations up to 3000 units in duration, the signals are reported to be un-analysable. (The typical period of oscillation for the parameters used is 20–25 time units.) Once a period has been determined, the network signals are analysed. Samples for a single period are generated and the traces of the individual cells compared, to group cells into classes executing the same behaviour with possibly differing phases. In all cases reported here, cells execute the same behaviour, that is they all exhibit the same voltage trace to the resolution of the grouping test (measured normalised trace correlation exceeds 0.95) though their phases within that common trace may vary. Once the cell behaviours have been grouped, the classification of oscillatory modes is performed. The analysis distinguishes the behaviours illustrated in Figure 2.

For a given choice of parameters, the network exhibits a number of oscillatory behaviours. In this study, we vary the two principal conductance parameters *g^el^* and *g^syn^*, over the range in which interesting behaviours occur, for networks comprising 2, 4 and 6 cells. The reported results generated as follows. For each pair of parameter values investigated:

1. a set of 8 random initial states for the network are generated, using a zero mean Gaussian distribution with 0.025 standard deviation. Random current input of length 250 time units is then constructed using independent identically Gaussian distributed random values with zero mean and 0.005 standard deviation for each 0.2 time unit step. For each set of initial conditions the model is integrated with the input current for around 10 periods. After this, input is removed and the behaviour of the network that results is then analyzed and classified.
2. the model is integrated from a zero initial state, and the generation of IP behaviour is checked. If IP is generated, random input current as described above is applied to see whether the IP is stable in the presence of noise. The behaviour is then classified.
3. the model is integrated from the zero initial state as before, and if stable IP behaviour is generated an attempt is made to switch the network to AP: half the cells receive a 1 unit positive current injection and half a 1 unit negative current injection for 0.2 time units, applied in successive tests at each time point between phase Φ = 0.4 and 0.6 in the cycle of oscillation. Again, random current inputs following the transient switching impulse are used to verify that the behaviour switched to is stable in the presence of noise. The behaviour is then classified.

The reported set of behaviours is the union of the results of the three steps above. This procedure, in our opinion, represents a reasonable compromise between computational effort and completeness of the results. Note, however, that it is not complete: the presence of any particular type of behaviour other than AP and IP is only detected if a suitable initial state is chosen (one that lies within the basin of attraction of that behaviour) and this, since only 8 initial states are generated at random, cannot be guaranteed.

### Firing rate model network

The mean field firing rate model of excitatory network activity with synaptic depression (formally equivalent to the relaxation oscillator model of a single cell described above) consists in the following equations (see [19]).
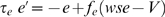


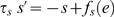
Here *e* represents network firing rate and varies between 0 (no activity) and 1 (all cells fire at their maximal frequency), *w* represents network connectivity, *s* is the synaptic depression variable which indicates the fraction of synapses available and *V* is the average firing threshold in the population. The sigmoidal function *f_e_(x)* = *1/(1+e^−x/ka^)* represents the input-output function of the network. Here the effective input equal to *wse−V* depends on activity *e* because of recurrent excitation. Activity depresses synapses as indicated by sigmoidal function *f_s_ (e)* = *1/(1+e^(e−θ)/ka^)* which decreases with *e*. Synaptic depression kinetics, determined by time constant *τ_s_*, is assumed to be slow compared to the network recruitment time constant *τ_e_*, so we are in a relaxation limit. The evolution of slow variable *s* produces switches between low and high activity states, thus the firing rate *e* undergoes periodic changes.

We then consider a network consisting of two such oscillatory units which are interconnected by cross-inhibition and electrical coupling (network scheme, Fig. 9), described as follows:
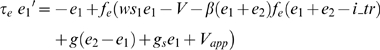


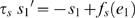


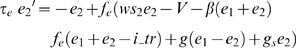


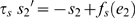
Here the input resulting from cross-inhibition depends on a sum *e_1_+e_2_* (since both excitatory units project to the same inhibitory population, on *f_e_(e_1_+e_2_−i_tr)* which models a threshold-like activation of the inhibitory population with threshold *i_tr* and on *β* which represents the strength of cross-inhibitory connectivity. To model electrical coupling between oscillatory units we first notice that the average membrane potential of a cell belonging to a given population is proportional to its firing rate *e*. Indeed, if *d_s_* is the duration and a_s_ is the voltage amplitude of a spike, whereas *d_is_* and *a_is_* are corresponding parameters characterized by inter-spike interval, then the mean voltage level *〈v〉*, averaged over *N* cycles expressed during time *T*, is equal to *(N/T)(d_s_a_s_+d_is_a_is_)*. Moreover, since *d_is_ = T/N−d_s_* we obtain: *〈v〉 = (N/T)(d_s_(a_s_−a_is_)+T/N_s_a_is_)* and therefore *〈v〉 = (N/T)d_s_(a_s_−a_is_)+a_is_*. Assuming *d_s_*, *a_s_* and *a_is_* do not depend on the firing rate, we obtain *〈v〉∼e*. Therefore the input resulting from electrical coupling between two different populations is equal to *g(e_2_−e_1_)* where *g* represents connectivity strength and the average difference between the spike amplitude and inter-spike voltage level. Recurrent electrical input is described by *g_s_e*, where *g_s_* represents the strength of recurrent connectivity. *V_app_* represents a brief input.
